# Supplementary material for: Preventive effects of nitrate-rich beetroot juice supplementation on monocrotaline-induced pulmonary hypertension in rats
Source: PLoS One. 2021 Apr 8;16(4):e0249816. doi: 10.1371/journal.pone.0249816 (PMC8031446; doi:10.1371/journal.pone.0249816)
Supplement: Attachment — Submitted filename: PLOS ONE (response).docx [file pone.0249816.s002.docx]

Responses to Reviewer #1

We are truly grateful for your careful reading of our manuscript and for your valuable comments. Our changes in the revised manuscript have been denoted in red. We hope our responses have sufficiently clarified the reviewers’ concerns.

- *The manuscript would be greatly strengthened by analysis of immune/inflammatory cells in the lung, either by flow cytometry or immunostaining or PCR.*

As you mentioned, this study did not address how BJ-derived nitrate prevents PH symptoms. Since this is a critical limitation of this study, a related description has been added in the Discussion (see line 308–317).

- *Is the proposed mechanism unique to the MCT model of PH or applicable to another etiological factor mediated PH? Have tried in another model of PH?*

We have never tried in another model of PH and there is also no report examining the effects of BJ supplementation on PH models other than MCT. Since dietary inorganic nitrate has been reported to be effective for hypoxia- and bleomycin-induced PH (Circulation. 2012; 125: 2922–2932), BJ supplementation might similarly have protective effects against PH models other than MCT. As you mentioned, this truth must be clarified in the future.

- *The authors have shown a preventative effect of BJ on development of PH. Translationally, how early would this need to be instituted to prevent or treat PH? Can PH be revered with BJ?*

Your comment points out a very important issue. Unfortunately, from our findings so far, we can only say that the intervention should start as soon as possible after the onset of PH. In addition, we reported that BJ supplementation is ineffective when started after PH had developed to some extent; however, only one dose was examined in that study (Am J Hypertens. 2019; 32: 216–222). That is, there is a possibility that a suitable amount of BJ (or nitrate) to reverse PH is different from that to prevent PH. Whether or not BJ supplementation can reverse PH is still under discussion.

- *It would be better to have a Figure with raw tracings of RVSP and the parameters (All together in one frame).*

According to your comment, typical tracings of RVSP have been added in the Figure (see Figs 1a and 3a).

- *Since right ventricle mainly affected in PH, any data to show the right ventricle pathology/collagen staining with picrosirius?*

In this study, we did not make paraffin blocks of right ventricle for histological analysis. Therefore, we are sorry but do not have any data to show morphological alterations of right ventricle.

- *BJ was given with water, suggest increase some discussion on BJ’s impact on gut or its microbiota if there is any possible axis in PH.*

As you mentioned, it cannot be denied that BJ supplementation may have been effective for PH by improving the intestinal environment. Therefore, according to your comment, a related description has been added in the Discussion (see line 312–316).

- *Suggest increased discussion of other possible indirect mechanisms, and any data refuting/suggesting them.*

According to your comment, a related description has been added in the Discussion (see line 274–278).

- *Was the lung inflated during the perfusion before the staining?*

Yes. We are sorry for our insufficient description and the corresponding part has been revised so that the readers can understand the exact procedure (see line 118).

- *A graphical abstract would be a great idea, showing the mechanisms of beneficial effects BJ?*

We agree your comment. However, this journal does not give an option to provide a graphical abstract. Therefore, we have added a summary scheme as the figure in the text (see Fig 5 and line 320).

Responses to Reviewer #2

We are truly grateful for your careful reading of our manuscript and for your valuable comments. Our changes in the revised manuscript have been denoted in red. We hope our responses have sufficiently clarified the reviewers’ concerns.

1. *Firstly, the study is not novel as a similar study has been done in humans “Effects of Oral Supplementation With Nitrate-Rich Beetroot Juice in Patients With Pulmonary Arterial Hypertension−Results From BEET-PAH, an Exploratory Randomized, Double-Blind, Placebo-Controlled, Crossover Study” by Henrohn et al. Oct 2018. Therefore, I would recommend the authors to expand on the current findings to explore additional parameters of endothelial function that may be impacted by beetroot supplementation.*

This study provides new evidence on the nitrate-dependent inhibitory effect of BJ on PH-induced pulmonary arterial remodeling. Although pulmonary arterial remodeling is an important index of PH, the report by Henrohn et al. did not address this issue. Therefore, we believe this study is of sufficient scientific novelty. As you mentioned, the influence of BJ supplementation on pulmonary endothelial function is very intriguing. Since we are familiar with experiments on evaluation of pulmonary vascular function (Am J Hypertens. 2020; 33: 305–309, Hypertens Res. 2020; 43: 178–185, J Pharmacol Sci. 2019; 140: 43–47, Life Sci. 2018; 203: 203–209), we are planning to perform such experiments. However, it takes a lot of time and animals to carry out the experiments, hence we would like to set those as the next theme. Thank you for your understanding.

2. *The authors also need to measure readouts of nitric oxide such as nitrate/nitrite levels post monocrotaline and beetroot administration in animals.*

According to your comment, data on plasma nitrite, nitrate, and their sum (NOx) levels and related descriptions have been added (see Figs 2 and 4, lines 32–34, 38–40, 102–103, 135–143, 179–191, 220–228, 245–248, 260–273, and 285–286). Although increases in those levels were not observed even by nitrate-rich BJ supplementation, we consider the fact that nitrate-depleted BJ was ineffective is the best evidence showing that BJ exerts beneficial effects in a nitrate-dependent manner.

3. *Histology images of pulmonary artery should be added to the manuscript.*

According to your comment, typical images of pulmonary artery have been added in the Figure (see Figs 1b and 3b).

4. *The experiments should have at least 5 replicates.*

In this study, it is the sham group that the sample size corresponds to less than 5. However, the sum of the first and second experiments gives 8 samples. In addition, we have collected a lot of sham data so far, and the sham data has little variation (Am J Hypertens. 2019; 32: 216–222, Life Sci. 2018; 203: 203–209). Based on these lines, we have decided that the sample sizes in the sham groups were acceptable limits. We believe our idea follows the 3R principles “replace/reduce/refine” for animal use for scientific purposes, and thank you for your understanding.

Responses to Reviewer #3

We are truly grateful for your careful reading of our manuscript and for your valuable comments. Our changes in the revised manuscript have been denoted in red. We hope our responses have sufficiently clarified the reviewers’ concerns.

- *The first experiment replicates previously published data with two doses of Beetroot juice. As previously published low dose beetroot juice is more effective in preventing PH than high dose beetroot juice. However, this interesting finding is not followed up.*

As you mentioned, it is an important issue why high-dose BJ supplementation was ineffective for MCT-induced PH. As described in the Discussion (see line 279–292), we think the reason for this lies in nitrate in BJ. However, this is difficult to prove because it cannot be demonstrated with nitrate-depleted BJ; unless PH is worsened by nitrate-rich BJ supplementation, it is of little significance to compare with its placebo. We do not know if it becomes a supplement to support the above hypothesis, but data on plasma nitrite, nitrate, and their sum (NOx) levels have been added (see Figs 2 and 4, lines 32–34, 38–40, 102–103, 135–143, 179–191, 220–228, 245–248, 260–273, and 285–286).

- *The second experiment focuses on nitrate depletion, and nitrate depleted beetroot juice is ineffective in reducing PH. However, no nitrate/nitrite plasma or urine levels are shown. Especially as there appears to be a dose dependent reaction to nitrate, it would be pertinent to the experiment to show systemic nitrate/nitrite levels in beetroot supplemented, depleted beetroot and naïve animals.*

According to your comment, data on plasma nitrite, nitrate, and NOx levels and related descriptions have been added (see Figs 2 and 4, lines 32–34, 38–40, 102–103, 135–143, 179–191, 220–228, 245–248, 260–273, and 285–286). Although increases in those levels were not observed even by nitrate-rich BJ supplementation, we consider the fact that nitrate-depleted BJ was ineffective is the best evidence showing that BJ exerts beneficial effects in a nitrate-dependent manner.

- *Pulmonary vascular remodelling is determined solely by measuring the medial thickness in chosen resistance pulmonary arteries. However the percentage of muscularised vessels is known to be increased in PH, and it would be helpful to this parameter (non, partial, fully muscularised percentages) when discussing effects on vascular remodelling.*

According to your comment, data on the percentage of remodeled vessels and related descriptions have been added (see Figs 1b and 3b, lines 30–31, 128–133, 154–155, and 196–197).

- *The paper is also lacking in mechanism. Does nitrate depletion lead to proliferation of SMC, or reduced inflammation in vessel walls or it purely a dilation effect of increased NO availability? Please add in some mechanistic studies.*

The results in this study show that nitrate is involved in the underlying mechanism by which BJ supplementation prevents PH development. Therefore, we believe this study has an aspect of the mechanistic study. However, as you mentioned, this study did not address how BJ-derived nitrate prevents PH symptoms. Since this is a critical limitation of this study, a related description has been added in the Discussion (see line 308–317).
