# Supplementary material for: Preventive effects of nitrate-rich beetroot juice supplementation on monocrotaline-induced pulmonary hypertension in rats
Source: PLoS One. 2021 Apr 8;16(4):e0249816. doi: 10.1371/journal.pone.0249816 (PMC8031446; doi:10.1371/journal.pone.0249816)
Supplement: Attachment — Submitted filename: PLOS ONE (reresponse).docx [file pone.0249816.s003.docx]

Responses to Editor

We are truly grateful for your careful reading of our manuscript and for your valuable comments. Our changes in the revised manuscript have been denoted in purple. We hope our responses have sufficiently clarified the editor’s concerns.

1) *It is important to evaluate the activity and protein expression of endothelial nitric oxide synthase (NOS) in lung tissue and pulmonary vessels in order to properly support the conclusions. Indeed, we don't have still evidence of pulmonary modulation of "good" NO production compared to "bad" NO production that depends on inducible NOS.*

Inorganic nitrate present in BJ produces NO in an eNOS-independent manner; therefore, we did not focus on the eNOS/NO pathway and did not measure eNOS activity and expression in this study. As you mentioned, excessive NO is a mediator of cytotoxicity in pulmonary circulation, but it is well known that PH is accompanied by a decrease in eNOS-derived NO bioavailability. Indeed, many studies have found that pulmonary eNOS activation by eNOS gene therapy (Am J Respir Cell Mol Biol. 2006; 35: 182–189, FASEB J. 2006; 20: 2594–2596) and chronic administration of L-arginine (Am J Physiol Endocrinol Metab. 2010; 298: E1131–E1139, Eur J Pharm Sci. 2014; 62: 161–170) and tetrahydrobiopterin (Cardiovasc Ther. 2018; 36: e12312) ameliorates the symptom of PH. Therefore, it is assumed that “good” NO production, regardless of endogenous generation or exogenous administration, is beneficial in the management of PH.

2) *The authors should better characterize cardiac phenotype by measuring cardiomyocyte size and myocardial collagen deposits.*

As mentioned in the response to the previous reviewers’ comments, we did not make paraffin blocks of right ventricle for histological analysis. Therefore, we are sorry but do not have data on cardiomyocyte size and myocardial collagen deposits.

3) *Previous study highlighted the importance of functional food compounds in cardioprotection through action on the epigenome (Eur Heart J. 2019 Feb 14;40(7):575-582). Since NO exerts epigenetic modifications, discussion on epigenetic regulation of expression of adaptive genes by NO should be added.*

According to your comment, a related description has been added in the Discussion (see lines 274–281).

Responses to Reviewer #3

We are truly grateful for your careful reading of our manuscript and for your valuable comments. Our changes in the revised manuscript have been denoted in purple. We hope our responses have sufficiently clarified the reviewer’s concerns.

- *Secondly they have added NOx plasma measurements as requested by multiple reviewers. The data show no significant changes in any NOx parameters in the LD BJ and the nitrate rich BJ supplementation groups. The only increases in plasma NOx is seen in the HD BJ group which paradoxically does not show any amelioration of PH. While it is reassuring that the experimental nitrate depleted control group shows no change from MCT alone, there is still insufficient evidence for mechanism. The authors suggest that it is local concentrations of nitrate rather than systemic that could be the driving factor for physiological effect but show no data to that effect. I feel adding lung NOx (and cGMP) concentrations in tissue would would add some mechanism to this paper.*

As you mentioned, we have not been able to address the issue of whether BJ supplementation caused a local increase in NOx levels. Although we agree with your comment that the data on lung NOx levels could provide some information, we did not keep lung tissues for NOx measurements. As the lack of tissue data is a critical limitation, this limitation has been further emphasized (see lines 316–319). However, even without this data, we think that our conclusion will be well supported by the results of experiments using nitrate-depleted PJ.

- *As noted the paper still lacks novel mechanistic insight. Previous studies by various groups referenced in this paper have suggested BJ /nitrate supplementation is effective though the NO-cGMP pathway affecting proliferation/dilation/endothelial function/inflammation etc. However there is no data in this paper to suggest that is what they are seeing.*

As described in the Introduction (see lines 46–54) and Discussion (see lines 321–322), there are already many reports showing the mechanism responsible for the protective effects of NO (or nitrate) on PH. Therefore, we did not focus on this in our study; the goal of this study was to determine a causative component for BJ-induced protective effects on PH (see lines 249–250). We think that the current data is sufficient to make a conclusion on this. In addition, we believe that our manuscript meets the publication criteria of PLOS ONE that “Authors may discuss possible implications for their results as long as these are clearly identified as hypotheses instead of conclusions”.

- *The authors have also suggested in the discussion that there could be effects on gut flora, but there is no attempt to include any data on that theory.*

A description regarding the gut flora was added based on the reviewer’s comment, and we do not have any data on the gut flora because we did not sample and store intestines and feces. Although the statement mentions that BJ supplementation might affect the intestinal environment, it does not imply that such a conclusion has been reached. Therefore, we do not think that our discussion is an overstatement.

- *The new schematic (Figure 5) is very general, and it would be better to add more specific pathways.*

What this study reveals is that BJ supplementation prevents the development of PH, including RVSP elevation, pulmonary arterial remodeling, and right ventricular hypertrophy, in a nitrate-dependent manner. As described in the Discussion (see lines 330–332), this finding is unprecedented and is not a generally-accepted one. In addition, this paper is not a review article; therefore, we have made a schema that summarizes only what can be said from this paper. As you mentioned, addition of more detailed information about specific pathways will be helpful, but we wonder if such a schema might have an exaggerated content. Thank you for your understanding.
